# The Real Experience and Management Strategies Analysis of Chinese Nurses Aiding COVID-19 Epidemic: A Qualitative Study

**DOI:** 10.1017/dmp.2020.477

**Published:** 2020-12-22

**Authors:** Linbo Li, Yongchao Hou, Fengying Kang, Suping Li, Juan Zhao

**Affiliations:** 1 Department of Mental Health, The First Hospital of Shanxi Medical University, Taiyuan, Shanxi, China; 2 Emergency Department, Shanxi Provincial People’s Hospital, Taiyuan, Shanxi, China; 3 Resident Standardized Training Central, The First Hospital of Shanxi Medical University, Taiyuan, Shanxi, China

**Keywords:** COVID-19 pneumonia, epidemic, nursing, work experience, qualitative study

## Abstract

**Objective:**

Emergent public health events, such as coronavirus disease 2019 (COVID-19), have been the focus of attention of researchers at home and abroad. In China, nurses are an important group contributing to the prevention and control of the COVID-19 pneumonia epidemic.

**Methods:**

Using semi-structured interviews, qualitative interviews were conducted with 23 nurses who supported the novel coronavirus pneumonia epidemic, and the data were collated and analyzed using Colaizzi analysis.

**Results:**

The work experience of Chinese nurses can be summarized into 4 major themes: they had different emotional experiences during aiding periods, aiding work had a double impact on the nurses, there were certain difficulties in aiding work, and there were significant age differences in aiding work experience.

**Conclusions:**

It is necessary to strengthen the psychological construction of nurses. All hospitals must coordinate and manage various safety tasks, and ensure the precise, scientific, and streamlined deployment of rescue work. Humanized management, shift adjustment, performance allocation weight, and organizational care are also the top priorities of human resource management.

Since December 2019, pneumonia caused by coronavirus disease 2019 (COVID-19) infection has been discovered in Wuhan, Hubei, China, and throughout the country. At present, the National Center for Disease Control has included it in the category of national Class B infectious diseases and taken measures for the prevention and control of Class A infectious diseases. Guo Yanhong, supervisor of the Medical Administration of the National Health Commission, introduced at the press conference of the Joint Defense and Joint Control Mechanism of the State Council on February 29, 2020, that 42,000 elite medical forces were sent nationwide, including 28,600 nurses, which accounts for 68% of the total number of medical teams. Embodying the noble spirit of blessing life, saving lives, and the willingness to give and love without bounds, they have become the main force in the fight against the epidemic.

With the development of the epidemic, the number of diagnosed and suspected patients is increasing, and the workload and work pressure of clinical front-line nurses in the fight against the epidemic have also increased.^1,2^ Chairman Wu Xinjuan pointed out that nurses were faced with not only heavy workloads and great psychological pressure, but also the risk of being infected in intensive care unit (ventilator care, airway management, emotional management, basic care, disinfection and isolation prevention, kidney replacement therapy). Previous studies^3^ mostly used the form of scales to assess the psychological state of clinical front-line staff, and few used qualitative research methods to dig deeply into the work experience and emotional needs of nursing staff during the major public health event periods. This study takes the COVID-19 pneumonia epidemic as an example to discuss the work experience of aiding clinical front-line nurses in depth.

Participants included nurses who supported the novel coronavirus pneumonia epidemic from January 22 to February 28, 2020. Inclusion criteria: age ≥18 y old; directly involved in clinical front-line work on epidemic rescue; obtained the nurse’s qualification certificate, volunteered and actively participated in the interview. Exclusion criteria: infection due to rescue work and there is a significant psychological stress disorder. A total of 23 nurses who worked in the epidemic were selected. A transcription analysis was also performed. Each participant was interviewed for approximately 30 min. Three people repeated the interview.

The interviews were based on the pre-set interview outline: Why did you choose to participate in COVID-19 epidemic?; What are your psychological feelings in the process of participating in the treatment of COVID-19 epidemic? Or what kind of emotional experience? If yes, please give examples; What impact do you think of participating in the epidemic prevention work on you?; What is the biggest difficulty you encountered in the process of clinical front-line rescue? How did you overcome these difficulties?; What do you think are the differences between you and your colleagues in rescue?; How do you deal with bad psychological feelings or difficulties?; Do you have any suggestions for managers in public health event prevention?

Data analyses used the qualitative research software MAXQDA12. Rigor was maintained through credibility, transferability, dependability, and confirmability. All the possible considerations and representations of study subjects were included to increase the credibility. Before conducting this study, we developed a trustworthy and friendly relationship with all participants. The researcher adhered to the study of theory and practice. Simultaneously, to ensure its validity during the research process, pre-interviews were conducted before beginning the study to improve the researcher’s practical grasp of interview techniques, analysis methods, and gradually improved the semi-structured interview outline with expert opinions.

## Results

In this study, the participants were 23 nurses, including 15 females (65.22%) and 8 males (34.78%), with an average age of 31.48 ± 2.30 y. In terms of educational level, 18 (78.26%) had a bachelor’s degree and 5 (21.74%) had a master’s degree. Regarding marital status, 12 (52.17%) are unmarried and 11 (47.83%) were married. Regarding duties, 5 were head nurses and 18 were nurses (see Table 1).

**Table 1.** General information of the participants (*n* = 23)

No.	Age (y)	Gender	Marital Status	Fertility Status	Education	Title	Working Age	Position
1	23	Female	Unmarried	Unbred	Bachelor	Nurse	1	Nurse
2	24	Male	Unmarried	Unbred	Bachelor	Nurse	1	Nurse
3	25	Male	Unmarried	Unbred	Bachelor	Nurse	1	Nurse
4	26	Male	Unmarried	Unbred	Bachelor	Nurse	1	Nurse
5	26	Male	Unmarried	Unbred	Bachelor	Nurse	1	Nurse
6	29	Female	Unmarried	Unbred	Master	Nurse practitioner	1	Nurse
7	34	Female	Married	Bred	Bachelor	Nurse-in-charge	9	Nurse
8	35	Female	Married	Bred	Bachelor	Nurse-in-charge	10	Nurse manager
9	36	Female	Married	Bred	Bachelor	Nurse-in-charge	12	Nurse
10	36	Female	Married	Bred	Master	Nurse-in-charge	12	Nurse manager
11	41	Female	Married	Bred	Bachelor	Nurse-in-charge	17	Nurse
12	45	Female	Married	Bred	Master	Associate professor of nursing	22	Nurse
13	50	Female	Married	Bred	Bachelor	Associate professor of nursing	30	Duty
14	23	Female	Unmarried	Unbred	Bachelor	Nurse	1	Nurse
15	24	Male	Unmarried	Unbred	Bachelor	Nurse	1	Nurse
16	25	Male	Unmarried	Unbred	Bachelor	Nurse	1	Nurse
17	26	Male	Unmarried	Unbred	Bachelor	Nurse	1	Nurse
18	26	Male	Unmarried	Unbred	Bachelor	Nurse	1	Nurse
19	29	Female	Unmarried	Unbred	Master	Nurse practitioner	1	Nurse
20	34	Female	Married	Bred	Bachelor	Nurse-in-charge	9	Nurse
21	35	Female	Married	Bred	Bachelor	Nurse-in-charge	10	Nurse manager
22	36	Female	Married	Bred	Bachelor	Nurse-in-charge	12	Nurse
23	36	Female	Married	Bred	Master	Nurse-in-charge	12	Nurse manager

Finally, after reviewing the literature and expert opinions, they were merged into three 4-level coding themes. The following 3 themes were identified: (1) they had different emotional experiences during the aiding period, such as (a) shock and heartache, (b) fear and anxiety, (c) excitement. (2) Aiding work had a double impact on the nurses: (a) positive effects included self-improvement, such as it has greatly improved in management skills, first-aid prevention and control systems, coordination and communication, and professional skills, and satisfaction, such as nurses believed that the professional care can be understood by the patients and supported by the leaders. (b) Negative effects included low sense of achievement, such as nurses sometimes felt deep lost because of the patient’s death and the ineffective treatment. In addition, there are certain occupational risks here, such as being infected. (3) There were certain difficulties in aiding work: (a) relatively insufficient medical resources, (b) uncertainty in the occurrence and development of the epidemic, and (c) significant age differences in aiding work experience. Young people have better pressure resistance, stronger perseverance, and a strong sense of responsibility than their predecessors. They want to help patients as much as possible to fight against death and the epidemic. The senior nurses pay more attention to infection prevention, scientific management, and first-aid skills.

## Discussion

During the period of support for the epidemic in Hubei, the nursing work environment is special and the risk is high. Nurses not only face the risk of COVID-19 infection, but also need to make a strong psychological stress response in a short time. At the personal level, we first actively encourage nurses to enhance their personal psychological potential and regulate negative emotions. On the 1 hand, some psychological methods, such as mindfulness breathing exercises, establishing close links with family members, watching videos, listening to music, talking to colleagues, and looking for favorable support systems, can effectively alleviate the psychological burden of aiding nurses.

Systems and laws are important guarantees to promote safety management. According to the provisions of the first consultation and accountability system in National Emergency Response Plan for Public Health Emergencies and the National Standards for the Management of Information Reports Related to Public Health Emergencies (for Trial), clarify job responsibilities, strengthen the concept of law, and respond to emergencies in accordance with the law.^4,5^ The medical staff across the country for COVID-19 pneumonia suggests that human resource management plays an important role in epidemic prevention and control. The human resources management during the epidemic includes personnel selection, team building, personnel training, and personnel use.^6^ Establish a “zero” infection target. At the stage of talent application, it is necessary to set up typical, benchmarking, and other forms to stimulate the nurses’ confidence, give full play to their internal potential, and enhance the social responsibility and sense of mission of aiding nurses.^7^ The COVID-19 pneumonia epidemic is facing a relative shortage of medical supplies. Managers should further standardize the procurement, storage, and deployment of materials for epidemic prevention and control to improve the material security capabilities.

The new generation of young nurses should have higher professional values. Nursing professional values is an important factor in determining the behavior of nursing staff. Nursing personnel play an important role in major public health emergencies. This requires nurses to have a strong interest in the cause of nursing and higher job satisfaction, and reduce the tendency of nursing staff to leave during the epidemic. The original intention is the best respect for nursing. At the same time, we need to cultivate a new generation of young nurses who love the nursing industry and have a good sense of social responsibility. The theme of the International Nurses’ Day for 3 consecutive years from 2017 to 2019 is “Nurse: Voice of Leadership.”^8^ In 2019, an article in *The Lancet* reported that the value of nursing is inestimable. It is proposed that nurses have great value and potential in responding to various crisis events, highlighting the importance of nurses in the development of human health in today’s society.^9^ However, this also means that nurses need to constantly improve themselves in the face of the whole career development plan and public health career, meet the challenges, and truly become adherents who have contributed to the health cause, and undertake greater social responsibility.

## Implications for Nursing Management

The implications of this study fell into 3 areas: —clinical practice, policy, and future research. For clinical practice, during the epidemic prevention and control period, it is necessary to strengthen the psychological construction of nurses and pay attention to the psychological quality education of nurses. All hospitals must coordinate and manage various safety tasks, and ensure the precise, scientific, and streamlined deployment of rescue work. For policy, humanized management, shift adjustment, performance allocation weight, and organizational care are also the top priorities of human resource management for epidemic prevention and control. It is also necessary to cultivate young nurses who love the nursing industry to make them have a good sense of social responsibility. Future research can observe the trajectory of emotional changes before and during nurse assistance during the new coronavirus epidemic, and the related needs of managers and management strategies in nursing work. Second, scientific nursing human resource management strategies and psychological nursing intervention research are urgently needed.
